# Assessment of the effectiveness of sustained release Bupropion and intensive physician advice in smoking cessation

**DOI:** 10.4103/0970-2113.59262

**Published:** 2010

**Authors:** Pranav Singh, Raj Kumar

**Affiliations:** Department of Respiratory Medicine, Vallabhbhai Patel Chest Institute, University of Delhi, Delhi-110007, India

**Keywords:** Smoking cessation, Bupropion quit rate, nicotine addiction

## Abstract

**Background:**

Tobacco use is the cause of immense burden on our nation in terms of mortality and morbidity, being the single leading cause of preventable illnesses and death. Smoking cessation interventions in our country will be the most cost effective of all interventions considering that the cost incurred on the three main tobacco related illnesses (COPD, CAD, and Cancer) being around Rs 27,761 crore in the year 1999.

**Matrials and Methods:**

A double blind placebo controlled trial was conducted to see the efficacy of Bupropion in smoking cessation. Smokers with current depression were excluded. The subjects (n = 30) were randomly assigned to receive Bupropion SR 300 mg/day or placebo for seven weeks. Target quit date was preferentially 8^th^ day of starting the treatment. Intensive counseling was provided by the physician at the baseline and brief counseling at every visit weekly during the treatment phase and at weeks 12 and 16. Self reported abstinence was confirmed by a carbon monoxide concentration in expired air of less than 10 ppm.

**Results:**

The seven-day point prevalence abstinence rate at the end of week 2 and week 16 in the drug group was 46.67% and 53.33 % respectively and in the placebo group was 13.33% and 20% respectively with the ‘*P*’ value of 0.04 and 0.05 respectively. Rates of continuous abstinence at weeks 4, 7 and 16 were 46.67%, 40% and 33.33% in the drug group and 13.33%, 13.33% and 13.33% in the placebo group respectively. The rates were significantly higher in the drug group till week 4 starting from week 2 of the treatment phase. The mean weight gain in drug group was found to be significant less as compared to the placebo at week 16 (*P* = 0.025) The mean change of depression scores from the baseline was not significantly different between the two groups at any point of time. The withdrawal symptom score increase from the baseline was not significantly higher at any point of time in the drug group but in the placebo group the increase was significantly higher for seven days after target quit date and at weeks 3 and 4 (*P* < 0.05). The most common adverse events in the drug group were insomnia, which was seen in 6 (40%) patients and dry mouth and/or altered taste in 4 (26.67%) patients, which was significantly higher as compared to placebo.

**Predictors of Outcome:**

The univariate predictors of a successful outcome were the point prevalence abstinence at week 16 were older age (>40 years), (*P* = 0.044) and quitter status at week 2 (*P* = 0.001). Multivariate predictors in order of importance were Quit status at 2nd week (*P* = 0.002) and Age >40 years (*P* = 0.031). The combined predictive value of these two variables was found to be 86.3%.

**Conclusions:**

Bupropion helps in smoking cessation. This has been proved by three large multicenter randomized controlled trials. This study has also reflected the same result in the form of significantly high seven-day point prevalence abstinence at week 16 in the Bupropion group as compared to placebo. Bupropion has a beneficial effect on weight gain and withdrawal symptoms and the benign adverse effects of insomnia and dry mouth or altered taste make it a very effective and cheap treatment for nicotine addiction in smokers.

## INTRODUCTION

The World Health Organization (WHO) estimates around 1.1 billion smokers worldwide constituting one-third of the entire population aged 15 years and above. 47% of the men and 12% of the women smoke globally.[1] It is predicted that by year 2020, tobacco will become the single leading cause of death, causing one in eight deaths.[2]

Tobacco use in India among the male and female population has been estimated at around 23.2% and 4% in urban and 33.6% and 8.8% in rural areas respectively.[3] The mortality burden of tobacco related deaths have been estimated at 800,000 deaths annually.[3] The burden of three main tobacco related illnesses i.e. COPD, coronary artery disease (CAD) and cancer is also immense. 4.2 million existing cases of CAD, 1,50,000 annual cases of cancer and about 12.5 million cases of COPD put an immense burden on the health infrastructure. The cost incurred on these three diseases is estimated at around Rs 27.761 crore in the year 1999.

The perceived benefits of tobacco industry like employment, GDP, export earnings are for the government while the health and financial costs are incurred by the users.[3] Some measures such as comprehensive tobacco control policies including taxation increase, consumer information, restriction on smoking at work places or public places can reduce tobacco consumption.[4]

In a country like India, where awareness levels are low, anti-tobacco education and medical help for those willing to quit will be the first step towards a tobacco free society.[3] Considering the social and economic impact of smoking, smoking cessation interventions are among the most cost effective of all medical interventions.[5]

Behavior conditioning and counseling were the main approaches available in early 80's before the availability of Nicotine replacement therapies (NRTs). Counseling and physician advice is effective and doctors should discuss it at every possible opportunity as alone it can help about 5% of the smokers to stop.

NRTs increase long term rates of smoking cessation and symptoms of nicotine withdrawal and users of NRTs are 1.5 – 2.7 times more likely to remain abstinent at one year as compared to the placebo.[5,6]

Bupropion SR is a non-nicotine containing treatment option which has been extensively tested in many clinical trials and its efficacy proven beyond doubt.[3] Majority of the studies on Bupropion have been done in the West. No study has been done on the Indian subjects. It would be ideal to combine the benefits of counseling and pharmacologic approach using Bupropion and perform a study to see its relevance in our country.

## MATERIALS AND METHODS

The study was conducted over a period of one year. Thirty patients were included in the study allocating 15 to the drug (Bupropion SR) and 15 to the placebo group. All the subjects were registered with the anti smoking clinic of Vallabhbhai Patel Chest Institute. A written consent was taken from all the patients. It was a single blind placebo control study. Inclusion criteria were, age ≥18 years, smokers ≥10 cigs/bidi per day for past one year, and subjects motivated to quit smoking. History of stroke or brain tumor, presence of an unstable cardiac or renal condition, pregnancy, lactation, current alcohol abuse (>2 “yes” on CAGE questionnaire), current use of a chewable tobacco containing product which the patient refuses to give up, current episode of major depression (Beck's depression inventory score of >9), and history of a current drug abuse were taken as excluding factors.

Baseline assessment of the patients was done to include the following parameters

1. Name, age, sex, marital status
2. Education, occupation, socioeconomic status
3. Detailed smoking history
4. Fagerström questionnaire: A measure of nicotine dependence with scores ranging from 0 to 11
5. Beck's depression inventory: 21 item self-administered questionnaire to assess the severity of depression. Scores of 0–9 taken as normal, 10–18 mild to moderate, 19–29 moderate to severe, 30–63 indicating severe depression
6. CAGE questionnaire a 4-point scale to screen for alcohol abuse
7. Physical examination
8. Laboratory investigations: hemoglobin, total Leukocyte count, differential leukocyte count, spirometry, ECG, chest roentgenogram
9. Body weight

### Treatment period

At the baseline, subjects were randomly assigned to two groups:

1. First group composed of patients who received physician advice based on National Cancer Institute's 5 A's i.e. ASK, ADVICE, ASSESS, ASSIST and ARRANGE. Sustain release Bupropion 300 mg was given as pharmacotherapy for seven weeks. This group had 15 subjects.
2. The second group composed of patients who received Physician advice based on National Cancer Institute's 5 A's i.e. ASK, ADVICE, ASSESS, ASSIST and ARRANGE. Sustain release Bupropion 300 mg was replaced by a placebo in this group. This group also had of 15 subjects.

### Follow-up period

Follow up assessment was weekly for the first seven weeks and then at week 8, 12 and 16. The subjects were telephoned three days after the target quit date and at subsequent due visit to remind them of the follow-up visits. Brief face-to-face personalized anti-smoking advice was given at each visit.

### Assessment at follow-up

1. Self reporting of abstinence was recorded at each visit
2. Exhaled CO measurement was done at each visit to validate the claim for abstinence in the past 24 hours
3. Daily diary was given to the patients to record the following data
  - Number of cig/bidi smoked per day
  - Medication (Bupropion SR) dose
  - Adverse events
  - Withdrawal symptoms (nine items)
    - Craving
    - Irritability
    - Anxiety
    - Depressed mood
    - Difficulty in concentrating
    - Restlessness
    - Increased appetite
    - Anger
    - Difficulty in sleeping
  Severity was assessed on a 5-point scale as absent (0), slight (1), mild (2), moderate (3) or severe (4). The symptoms were recorded daily by the patients for the first week after target quit date and then weekly at the follow-up for five weeks.
4. Body weight was recorded at seven and 16 weeks
5. ECG at the end of the treatment phase at week 7
6. Beck's depression inventory: Scores repeated at week 7 and 16.

### Measures of outcomes

1. Seven days point prevalence: Measured as absence of smoking for past seven days. To be done weekly for seven weeks, then at eight, 12 and 16 weeks. Self reporting of abstinence was validated by breath CO monitoring (<10 ppm taken as acceptable proof of abstinence for past 24 hours).
2. Continuous abstinence: All the participants meeting the abstinence criteria (not even a single puff) at every visit will be considered continuously abstinent. Measured weekly during treatment phase, at week 12 and 16.
3. Withdrawal symptoms: Measured as change from baseline.
4. Beck depression scores: Measured as change from baseline.
5. Body weight: Measured as change from baseline.
6. Predictors of successful outcome.

### Statistical analysis

The baseline characteristics of the two groups were compared by analysis of variance for continuous variables and Chi square test for categorical variables [Table 1]. The efficacy of smoking cessation was evaluated with the use of weekly point prevalence abstinence rates and rates of continuous abstinence. Subjects who missed a follow-up, discontinued treatment or were lost to follow up were considered to be smoking. The Chi Square analysis was used to determine the difference between these rates in two groups at various points of time. Withdrawal symptoms were assessed at baseline, daily for the first week after target quitting date and weekly at weeks 3, 4, 5, 6 and 7. A composite withdrawal score was calculated as a mean of nine withdrawal symptoms. The mean withdrawal score in each group at the various points of time was compared to the baseline by repeated measures analysis of variance. Body weight and symptoms of depression were assessed in subjects who completed 16 weeks of follow up. It was measured at baseline, week 7 and week 16. Repeated measure analysis of variance was used to assess the change. The rates of adverse events in two groups were compared with Chi Square analysis. Predictors of outcome were studied using Univariate analysis and a step wise multiple logistic regression analysis to study the multivariate predictors of quitting.

**Table 1:** Baseline characteristics of the two groups

Characteristic	Drug group n = 15	Placebo group n = 15	*P* value
Age (in years)	46.87 ± 14.12	39.33 ± 12.18	0.129
Sex (%)			1.00
Males	100	93.33%	
Females	0	6.67	
Body weight (kg)	67.27 ± 13.77	70.07 ± 15.09	0.6
Marital status (%)			0.598
Married	93	80	
Education (%)			0.086
Illiterate	6.67	0	
Primary to intermed	20	60	
Graduate	60	40	
Professional	13.33	0	
Occupation (%)			0.183
Professional/business	60	46.67	
Clerical	0	0	
Manual	6.67	26.67	
Student	6.67	20	
Unemployed	26.67	6.67	
Economic status (Rs.)			0.301
Lower (<3000)	13.33	0	
Middle (3000–10000)	26.67	40	
Higher (<10000)	60.0	60	
Age at start of smoking (mean ± sd)	20.73 ± 5.54	20.80 ± 6.43	0.461
Years of smoking (means ± sd)	26.13 ± 14.22	20.47 ± 13.33	0.269
Type of product (%)			1.00
Cigarette	73.33	66.67	
Bidi	26.67	33.33	
Cig/bidi per day in past 1 year	19.47 ± 6.86	18.13 ± 6.05	0.577
Money spent per day in Rs (mean ± sd)	26.13 ± 16.92	20.13 ± 14.10	0.300
Previous quit attempts (%)			0.700
Yes	73.33	60	
No	26.67	40	
No. of attempts (mean ± sd)	2.600 ± 0.8552	2.600 ± 1.013	1.00
Method used for abstinence (%)			
Counselling	100	100	NA
Cold turkey			
Drugs			
Family history (%)			1.00
Present	46.66	40	
Absent	53.33	60	
Awareness of harmful effects (%)			0.483
Yes	100	86.67	
No	0	13.33	
Awareness of ban (%)			0.483
Yes	86.67	100	
No	13.33	
Fagerström score (mean ± sd)	5.80 ± 1.9	5.47 ± 2.6	0.665
Beck depression scores (mean ± sd)	6.73 ± 1.94	6.20 ± 2.6	0.529

NA = Data not available

## RESULTS

The baseline characteristics of two groups are as shown in Table 1. The mean age of patients in the drug group and the treatment group was 46.87 ± 14.12 and 39.33 ± 12.18 respectively, suggesting that the middle to elderly age group is more likely to seek help for smoking cessation. There was only one female patient among a total of 30 patients seeking anti smoking advice. The percentage of married smokers was significantly higher (*P* < 0.05) in both groups although there was no difference between the two groups.

Except one illiterate patient, almost all the patients in the two groups had at least primary level education (*P* < 0.05) although there was no difference between the two groups in educational status. Almost 50% of the patients were either professionally employed or in business in both the groups, with a small percentage of manual labor, unemployed or students. Twenty eight out of 30 (93.33%) patients belonged to the middle or the higher income group with no statistical difference between the groups.

Mean age of onset of smoking in the drug group was 20.73 ± 5.54 and that in the placebo group was 20.80 ± 6.43, signaling towards a young age of onset in both the groups without any significant difference between the two groups. Mean number of years of smoking was 26.13 ±1 4.22 in drug group and 20.47 ± 13.33 in the control group with no difference between the groups. 73.33% in drug group and 66.67% in the control group were cigarette smokers and the rest were bidi smokers although there was no difference between the groups. The number of cigarettes or bidis smoked per day in the past one year was 19.46 ± 6.86 and 18.13 ± 6.05 in drug and control group respectively with no significant difference between the two groups.

Rs 26.13 ± 16.92 per day was spent by the drug group and Rs 20.13 ± 14.10 was spent by the control group in purchasing products related to smoking with no difference between the groups. About 73.33% of patients in the drug group had made a mean number of 2.6 ± 0.8 number of quit attempts and 60% in the placebo group had a mean number of 2.6 ± 1.013 number of quit attempts with no difference between the groups.

Hundred per cent of the patients who had tried to quit in the past did so on their own i.e. cold turkey without any assistance from the physician and none of them used any medication to quit. Family history of smoking was present in almost half of all the patients without any significant difference between the two groups. 100% of patients in drug group and 86.67% in the control group were aware of at least one harmful effect of smoking and 86.67% in the former and 100% in the latter were aware of the ban on smoking in public places and the awareness level was the same in two groups.

The mean Fagerström score in drug group was 5.80 ± 1.9 and that in the control group was 5.47 ± 2.6 without any difference between the two groups. The Beck depression inventory scores were 6.73 ± 1.94 and 6.20 ± 2.6 in the drug and control groups respectively with no significant difference between the two groups.

### Seven-day point prevalence abstinence rates

**Figure 1:**
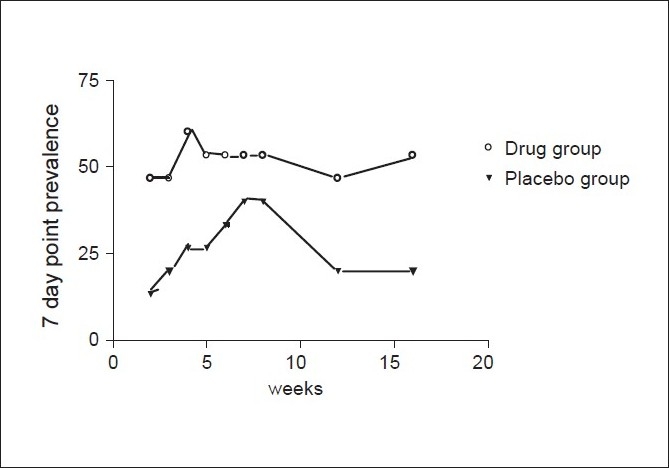
Seven-day point prevalence rates (% of subjects)

These are shown in the Table 2 every week for seven weeks after the target quit date and at week 12 and 16. The seven-day point prevalence rates, as seen in Figure 1, show persistently higher trends in the drug group although the rates showed a statistical significance in weeks 2 and 16 (*P* ≤ 0.05).

**Table 2:** Point prevalence rates starting from week 2 of the treatment phase

Week of study	Drug group (point prevalence) (%)	Placebo group (point prevalence) (%)	*P* value
2	7/15 (46.67)	2/15 = 13.33	0.04*
3	7/15 (46.67)	3/15 (20)	0.12
4	9/15 (60)	4/15 (26.67)	0.06
5	8/15 (53.33)	4/15 (26.67)	0.13
6	8/15 (53.33)	5/15 (33.33)	0.26
7	8/15 (53.33)	6/15 (40)	0.46
8	7/15 (53.33)	3/15 (40)	0.12
12	7/15 (46.67)	3/15 (20)	0.12
16	8/15 (53.33)	3/15 (20)	0.05*

* denotes statistical significance

### The continuous abstinence rates

**Figure 2:**
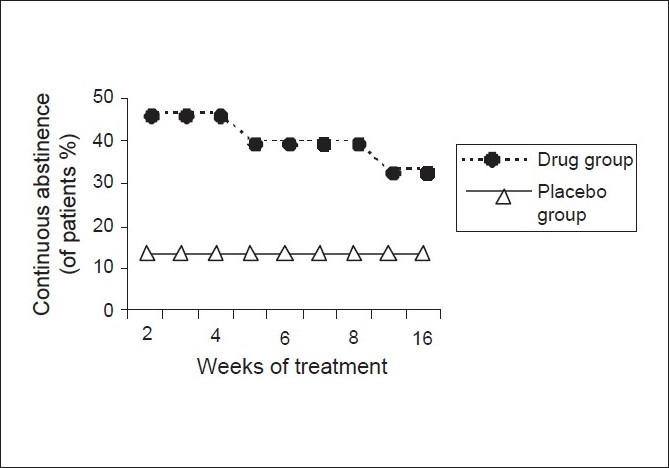
Comparison of continuous abstinence rates between two groups

The continuous abstinence rates (% of abstinent patients at each visit) are depicted in Table 3. As seen from Figure 2, they are persistently higher in the drug group as compared to the placebo although the results reached statistical significance at weeks 2, 3 and 4 of the treatment phase. (*P* ≤ 0.5). The rates at weeks 5, 6, 7 and 8 had *P* value of (0.05 < *P* < 0.1)

**Table 3:** Continuous abstinence rates starting week 2 of the treatment phase

Week of study	Drug group continuous abstinence (%)	Placebo group continuous abstinence (%)	*P* value
2	7/15 (46.67)	2/15 (13.33)	0.04*
3	7/15 (46.67)	2/15 (13.33)	0.04*
4	7/15 (46.67)	2/15 (13.33)	0.04*
5	6/15 (40)	2/15 (13.33)	0.09
6	6/15 (40)	2/15 (13.33)	0.09
7	6/15 (40)	2/15 (13.33)	0.09
8	6/15 (40)	2/15 (13.33)	0.09
12	5/15 (33.33)	2/15 (13.33)	0.19
16	5/15 (33.33)	2/15 (13.33)	0.19

* denotes statistical significance

### Body weight

**Figure 3:**
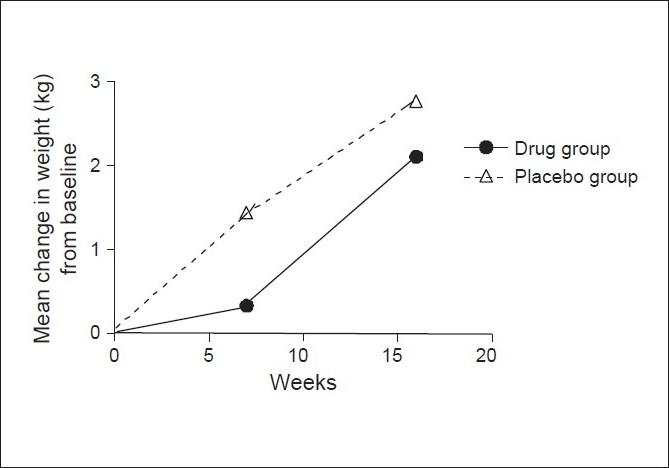
Mean change in weight from baseline at weeks 7 and 16 in drug group and placebo

This was measured at baseline, at week 7 and week 16. The means are tabulated in the Table 4. There was a mean gain of 2.11 kg in the drug group and 2.77 kg in the placebo group at 16 weeks as compared to the baseline. A linear trend in weight gain starting from the baseline was seen in both the groups with *P* =.002 as shown in Figure 3.

**Table 4:** Mean weights in two groups at three points of time

Week	Mean wt in drug group (kg)	Mean wt in placebo group (kg)
0	60.11 ± 8.98	74.56 ± 15.08
7	60.44 ± 9.13	76.00 ± 15.81
16	62.22 ± 10.72	77.73 ± 16.00

The weights were found to be significantly high at 16 weeks as compared to baseline in each group (*P* = 0.016). Comparing the mean change in body weight from the baseline at week 16 between the two groups showed that the increase in weight in the drug group was significantly less than that in the placebo group (*P* = 0.025).

### Depression scores

**Figure 4:**
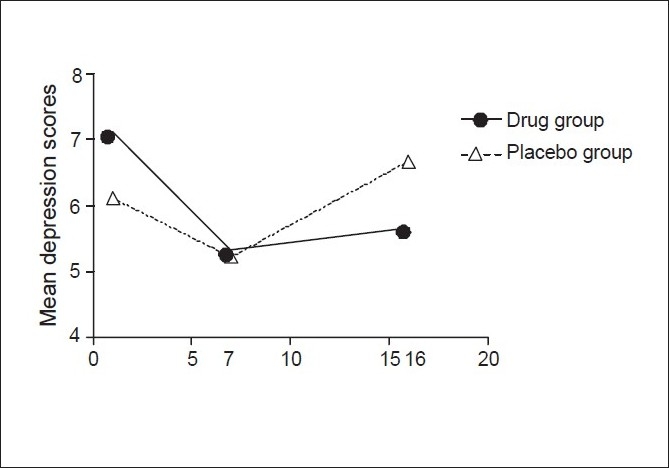
Comparison of mean depression scores in two groups

The mean depression scores, as measured by the Beck's depression inventory, in the drug group and treatment group are shown in Table 5. The mean depression scores showed a decrease from the baseline at week 7 in both the drug and placebo group as seen in Figure 4, but the fall reached statistical significance only in the drug group at week 7 (*P* = 0.51). The mean change from the baseline did not differ between the drug group and the placebo group at weeks 7 and 16.

**Table 5:** Mean BDI scores in each group

Week	Mean BDI score in drug group (kg)	Mean BDI score in placebo group (kg)
0	7.11 ± 1.76	6.11 ± 3.02
7	5.33 ± 1.87	5.22 ± 2.82
16	5.67 ± 2.87	6.67 ± 2.84

As seen in Figure 4, the mean depression scores had a significant quadratic trend in both the groups (*P* = 0.003) thereby implying a fall in the scores at 7 weeks followed by a rise at 16 weeks.

### Withdrawal scores

**Figure 5:**
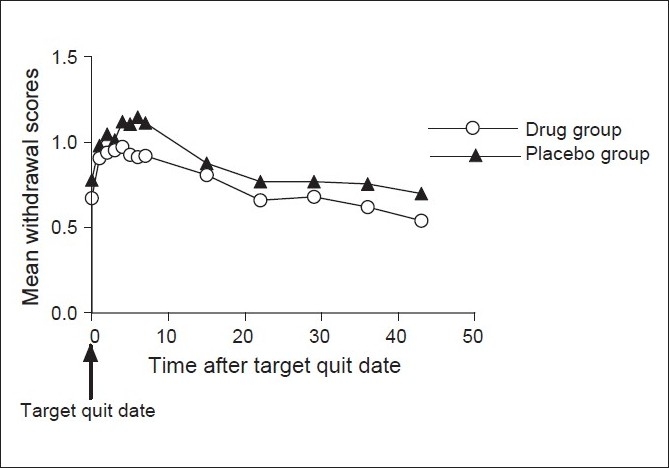
Mean withdrawal scores in the two groups starting from the target quit date

The baseline withdrawal scores were recorded in both the groups daily for seven days after the target-quit date and at weeks 3, 4, 5, 6 and 7 of the treatment phase. Table 5 shows the mean withdrawal scores for each group at various points of time. The mean withdrawal scores, as seen in Figure 5, increased from the baseline in both the drug group and the placebo group in the first seven days and at week 3 of treatment period after the target quit date. In the drug group, the mean increase from the baseline did not reach statistical significance at any point of time (*P* > 0.05). In the placebo group, the mean withdrawal scores were significantly higher as compared to the baseline at days 1,2,3,4,5,6,7,after target quit date and at weeks 3 and 4 of the treatment period (*P* < 0.05) [Table 6].

**Table 6:** Mean withdrawal scores at various points of time in two groups

(Time of measurement (after target quit date)	Mean withdrawal score (drug group)	Mean withdrawal score (placebo)
Baseline	0.673 ± 0.499	0.780 ± 0.689
Day		
1	0.907 ± 0.590	0.980 ± 0.713
2	0.940 ± 0.632	1.047 ± 0.715
3	0.953 ± 0.646	1.013 ± 0.756
4	0.973 ± 0.696	1.120 ± 0.778
5	0.927 ± 0.703	1.1070 ± 0.750
6	0.913 ± 0.742	1.147 ± 0.787
7	0.920 ± 0.761	1.113 ± 0.733
Week		
3	0.808 ± 0.719	0.877 ± 0.596
4	0.66 ± 0.67	0.77 ± 0.64
5	0.68 ± 0.70	0.77 ± 0.80
6	0.620 ± 0.618	0.756 ± 0.770
7	0.54 ± 0.49	0.70 ± 0.67

### Adverse events

**Figure 6:**
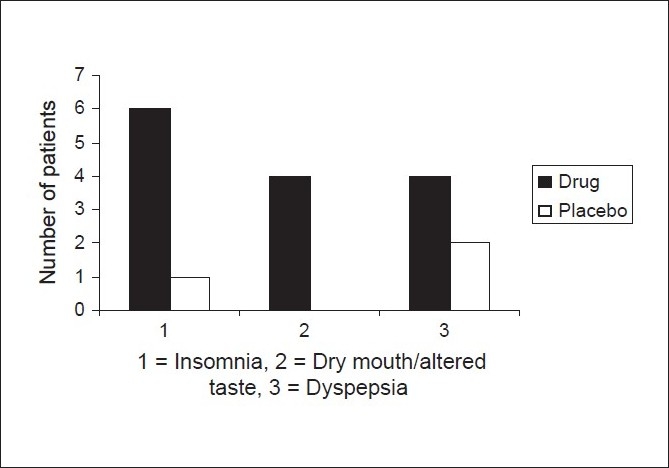
Comparison of adverse events in the drug and placebo group

Insomnia was seen in six (40%) of the patients in the drug group and one (6.67%) patient in the control group (*P* = 0.031). Four patients, exclusively in the drug group, complained of sensation of altered taste and or dry mouth with no such complaint coming from the placebo group (*P* = 0.038) [Figure 6].

One patient in drug group had loss of appetite during the treatment phase. Dyspepsia manifested as bloating and distension was seen in four patients in drug group and two patients in control group without any significant difference between the groups. One patient in the drug group had an episode of left sided chest pain and had non-specific ST-T wave changes on ECG. Detailed cardiovascular evaluation did not reveal any abnormality.

### Predictors of outcome (univariate predictors)

The parameters studied to gauge their effect on the successful outcome, which was the quitter status at week 16 (dependent variable), are shown in Table 7.

**Table 7:** Univariate predictors of outcome

Factor	No. in quitter group	No. in non quitter group	*P* value
Age			0.044*
20–39	2	11	
40–59	4	6	
≥60	5	2	
Marital status			0.268
Married	11	15	
Unmarried	0	4	
Education			0.835
Illiterate	0	1	
Prim to inter	4	8	
Graduate and above	6	9	
Professional	1	1	
Eco status			0.866
Lower (<3000)	1	1	
Middle (3000–10000)	4	6	
>10000	6	12	
Age at start			0.142
<20	6	5	
20–30	5	10	
>30	0	4	
Type of product			1.00
Cigarette	8	13	
Bidi	3	6	
No. of bidi/cig			0.858
≤15	4	7	
16–25	6	9	
>25	1	3	
Previous attempts			1.00
Yes	7	13	
No	4	6	
Number of attempts			0.698
0	4	6	
1–5	6	9	
6–10	1	4	
Family history			0.858
Yes	5	8	
No	6	11	
Fagerstr�m score			0.246
1–6	9	11	
≥7	2	8	
Depression score			0.244
0–4	2	5	
5–9	9	14	
Quit status at 2^nd^ week			0 .001*
Yes	8	2	
No	3	17	
Clinical symptoms			0.705
Present	6	9	
Absent	5	10	
Airway obstruction			0.687
Present	4	5	
Absent	7	14	

Multivariate predictors of quitting using a stepwise multiple logistic regression model were:

1. Quit status at 2^nd^ week: Those who had quit at week 2 of the treatment phase were most likely to be abstinent at week 16 (*P* = 0.002) (step 1).
2. Age: Patients in 20 to 39 year age group were least likely to quit at 16 weeks or in other words age >40 years is associated with quitting at 16 weeks (*P* = 0.031) (Step 2).

Presence of these two variables can predict whether a person will continue to abstain at 16 weeks with an accuracy of 83.3%.

## DISCUSSION

The study was carried out on motivated patients attending the anti smoking clinic at Vallabhbhai Patel Chest Institute, New Delhi over a period of one year. The baseline characteristics of the patients did not differ significantly. Except for one female in the placebo group, all the patients in both the groups seeking help for smoking cessation were males despite a considerable percentage (about 6%) of female smokers in urban areas. This highlights the fact that the female coming out with their smoking habit is considered a social stigma and this problem needs to be addressed if they are to benefit from the tobacco cessation interventions.[7]

According to a study done to determine the patterns of smoking in Delhi, smoking was most prevalent among the illiterates.[7] In the present study only two of the 30 patients seeking help were illiterates, thus making this group least likely to seek help as compared to those with a minimum of elementary education. There is a need for tobacco cessation programs to target this group so that it gets its fair share of opportunity in participating in such programs. Most of the smokers seeking help (>50%) were either professionals, or self-employed belonging to middle or the higher income group.

Although bidi is the most common tobacco product smoked in India yet only 30 percent of smokers in the present study were bidi smokers and cigarette smokers formed the rest 70%.

All the smokers who had a prior quit attempt did so on their own without any help from the physician or use of any medication that is all tried quitting cold turkey. This is very important as physician advice regarding quitting is not been given as frequently as it should be and anti smoking advice as part of patient management is still provided rarely, this in spite of the fact that five per cent of smokers will quit only on their physicians advice.[4]

Awareness regarding the harmful effects and the ban on smoking at public places was found to be quite high exceeding 90% in both the groups. This could be attributed to the fact that except two illiterate smokers, all the smokers have had at least primary level of education.

The seven-day point prevalence abstinence rate at the end of week 2 in the drug group was 46.67% and in the placebo group was 13.33% and former being significantly higher than the latter (*P* = 0.04). The rates at the end of treatment phase i.e. week 7 and at weeks 8, 12, 16 were 53.33%, 33.33%, 46.67% and 53.33% in drug group and 40%, 40%, 20% and 20% in placebo group respectively. The rates were higher in the drug group at all points of time but reached statistical significance only at week 16 (*P* = 0.05). The seven-day point prevalence rate in various studies has ranged from 32 to 50% with Bupropion and the observed seven-day point prevalence abstinence rate of 53.33% in this study is in line with those studies.[3,8–10]

Rates of continuous abstinence at weeks 4, 7 and 16 were - 46.67%, 40% and 33.33% in the drug group and 13.33%, 13.33% and 13.33% in the placebo group respectively. The rates were significantly higher in the drug group till week 4 starting from week 2 of the treatment phase. The continuous abstinence rate of 33.33% at week 16 in the drug group was more than twice that in the placebo group i.e. 13.33%.

These rates are comparable to the studies where continuous abstinence rates at week 7 ranged from 24 to 35 % while those at week 16 ranged from 22% to 35%.[3,8–10]

The mean weight gain in the drug group was 2.11 kg while that in the placebo group was 2.77 kg. The weight gain in both the groups was significantly higher as compared to baseline at weeks 7 and 16. The mean weight gain in drug group was found to be significant less as compared to the placebo at week 16 by using the unpaired *t* test (*P* = 0.025). This is in line with various studies reporting beneficial effects of Bupropion on weight gain in the post cessation period.[3]

The depression scores decreased from the baseline in both the groups and at week 7 the mean depression score in the drug group (5.33 ± 0.87) was significantly less than the baseline (7.11 ± 1.76). The mean change of depression scores from the baseline was not significantly different between the two groups at any point of time. This is in agreement with the studies wherein Bupropion SR was found not to have a significant beneficial effect on depression scores over the placebo.[3]

Withdrawal symptom scores increased from baseline in both the groups at all points of time in the post cessation period. The increase from the baseline was not significantly higher at any point of time in the drug group but in the placebo group the increase was significantly higher for 7 days after target quit date and at weeks 3 and 4 (*P* < 0.05). This highlights the beneficial effect of Bupropion in reducing the withdrawal symptoms as compared to the placebo and hence helps the patient in the quitting process. It has been brought out by various studies that Bupropion reduces the withdrawal symptom scores significantly better than placebo and NRT.[8–10]

The most common adverse events in the drug group were insomnia, seen in 6 (40%) patients and dry mouth and/or altered taste in 4 (26.67%), which was significantly higher compared to placebo. These two adverse events are also the commonest reported in the literature.[3] Dyspepsia was seen in 4 (26.67%) patients and has not been reported elsewhere as an adverse effect of Bupropion, though it was not significantly different from the placebo group where it was seen in two patients.

The univariate predictors of successful outcome which was the point prevalence abstinence at week 16 were older age (>40 years), (*P* = 0.044) and quitter status at week 2 (*P* = 0.001). Multivariate predictors of a successful outcome were studied using a stepwise multiple logistic regression model. The variables in order of importance were quit status at 2^nd^ week (*P* = 0.002) and age>40 years (*P* = 0.031). The combined predictive value of these two variables was found to be 86.3%. In other words if a smoker had quit at second week of treatment he was most likely to remain abstinent till 16^ th^ week. Similarly older age (>40 years) also predicted that patient had significant chances of quitting and remaining abstinent till the 16^th^ week. Other predictors of outcome in studies on Bupropion such as lower smoking rates, periods of abstinence, previous quit attempts, and male gender were not found to be significant in present study.[11]

The main limitation of the study was a small 'number of patients (n)' as the 'n' required to detect a significant difference in abstinence rates between Bupropion group and placebo group at a nominal *P* of 0.05 and power of 80% based on stipulated abstinence rates in previous studies was found to be at least 400 and such a large 'n' was not possible in the present study considering the time constraint of one year and a long follow up of 16 weeks.

## CONCLUSIONS

The study was done on smokers attending the anti smoking clinic. Detailed smoking and clinical history was recorded, Fagerström test for nicotine dependence was administered and Beck depression inventory scores were obtained. Relevant clinical examination and investigations were performed. Patients were randomly divided into two groups. One group received drug (Bupropion) and the other received the placebo after a detailed counseling.

Bupropion helps in smoking cessation. This has been proved by three large multicentre randomized controlled trials.[8–10] Our study has also reflected the same result in the form of significantly high 7-day point prevalence abstinence at week 16 in the Bupropion group as compared to placebo. Bupropion has a beneficial effect on weight gain and withdrawal symptoms and the benign adverse effects of insomnia and dry mouth or altered taste make it a very effective and cheap treatment for nicotine addiction in smokers.
